# Supplementary material for: End Sequence Analysis Toolkit (ESAT) expands the extractable information from single-cell RNA-seq data
Source: Genome Res. 2016 Oct;26(10):1397–410. doi: 10.1101/gr.207902.116 (PMC5052061; doi:10.1101/gr.207902.116)
Supplement: Supplemental Material [file supp_gr.207902.116_Supp_Materials.tar.gz › ESAT-esat_v0.1/doc/html/classbroad_1_1pda_1_1feature_1_1genome_1_1_directory_installed_genome_assembly-members.html]

ESAT: Member List


ESAT


- Main Page
- Related Pages
- Packages
- Classes
- Files

- Class List
- Class Index
- Class Hierarchy
- Class Members

All Classes Namespaces Files Functions Variables Enumerator Pages

- broad
- pda
- feature
- genome
- DirectoryInstalledGenomeAssembly

broad.pda.feature.genome.DirectoryInstalledGenomeAssembly Member List

This is the complete list of members for broad.pda.feature.genome.DirectoryInstalledGenomeAssembly, including all inherited members.

|  |  |  |
| --- | --- | --- |
| areRepeatsInDeepDirHierarchy() | broad.pda.feature.genome.DirectoryInstalledGenomeAssembly | protected |
| delete(SequenceRegion region) | broad.pda.feature.genome.DirectoryInstalledGenomeAssembly |  |
| DirectoryInstalledGenomeAssembly(File sequenceDirectory) | broad.pda.feature.genome.DirectoryInstalledGenomeAssembly |  |
| doRepeatListNeedsCleanup() | broad.pda.feature.genome.DirectoryInstalledGenomeAssembly | protected |
| drawAutosome() | broad.pda.feature.genome.DirectoryInstalledGenomeAssembly |  |
| drawChromosome() | broad.pda.feature.genome.DirectoryInstalledGenomeAssembly |  |
| drawRandomAutosomeRegion(int size) | broad.pda.feature.genome.DirectoryInstalledGenomeAssembly |  |
| drawRandomRegion(int size) | broad.pda.feature.genome.DirectoryInstalledGenomeAssembly |  |
| getAllChromosomes() | broad.pda.feature.genome.DirectoryInstalledGenomeAssembly |  |
| getAllNonRandomChromosomes() | broad.pda.feature.genome.DirectoryInstalledGenomeAssembly |  |
| getAnnotationDirectory() | broad.pda.feature.genome.DirectoryInstalledGenomeAssembly |  |
| getAutosomeGenomeSize() | broad.pda.feature.genome.DirectoryInstalledGenomeAssembly |  |
| getAutosomeNumber() | broad.pda.feature.genome.DirectoryInstalledGenomeAssembly |  |
| getAutosomes() | broad.pda.feature.genome.DirectoryInstalledGenomeAssembly |  |
| getChromosome(String chr) | broad.pda.feature.genome.DirectoryInstalledGenomeAssembly |  |
| getGenomeSize() | broad.pda.feature.genome.DirectoryInstalledGenomeAssembly |  |
| getNonRandomTotalSize() | broad.pda.feature.genome.DirectoryInstalledGenomeAssembly |  |
| getReducedRepresentation(int totalSize, List< GenomicAnnotation > toAvoid) | broad.pda.feature.genome.DirectoryInstalledGenomeAssembly |  |
| getRepeatFile(String chrSymbol) | broad.pda.feature.genome.DirectoryInstalledGenomeAssembly |  |
| getSequenceDir() | broad.pda.feature.genome.DirectoryInstalledGenomeAssembly |  |
| getUn() | broad.pda.feature.genome.DirectoryInstalledGenomeAssembly |  |
| getX() | broad.pda.feature.genome.DirectoryInstalledGenomeAssembly |  |
| insert(SequenceRegion region, GenomicAnnotation insertionPoint) | broad.pda.feature.genome.DirectoryInstalledGenomeAssembly |  |
| invert(SequenceRegion region) | broad.pda.feature.genome.DirectoryInstalledGenomeAssembly |  |
| sequenceDirectory | broad.pda.feature.genome.DirectoryInstalledGenomeAssembly | protected |
| translocate(SequenceRegion region1, SequenceRegion region2) | broad.pda.feature.genome.DirectoryInstalledGenomeAssembly |  |
| useMergedUn() | broad.pda.feature.genome.DirectoryInstalledGenomeAssembly | protected |
| writeSizes(String outFile) | broad.pda.feature.genome.DirectoryInstalledGenomeAssembly |  |


---

Generated on Fri Aug 15 2014 15:10:59 for ESAT by  

 1.8.7
